# Comparison of Clinical Estimation and Stereophotogrammic Instrumented Imaging of Burn Scar Height and Volume

**DOI:** 10.3390/ebj5010004

**Published:** 2024-02-12

**Authors:** Shyla Kajal Bharadia, Vincent Gabriel

**Affiliations:** 1 Cumming School of Medicine, University of Calgary, 2500 University Dr. NW, Calgary, AB T2N 1N4, Canada; shyla.bharadia@ucalgary.ca; 2 Departments of Clinical Neurosciences and Surgery, Faculty of Medicine, University of Calgary, 2500 University Dr. NW, Calgary, AB T2N 1N4, Canada; 3 Calgary Firefighters’ Burn Treatment Centre, Foothills Medical Centre, 1403-29 Street NW, Calgary, AB T2N 2T9, Canada; 4 McCaig Institute for Bone and Joint Health, Faculty of Medicine, University of Calgary, 2500 University Dr. NW, Calgary, AB T2N 1N4, Canada

**Keywords:** outcome measure, burn, scars, stereophotogrammetry

## Abstract

Descriptive clinical tools for characterizing burn scars are limited by between-user variability and unknown sensitivity to change over time. We previously described preclinical assessment of stereophotogrammetry as a valid measure of burn-related scars. Here, we compared the estimated vs. instrumented measurements of maximum height and total positive volume of 26 burn scars. The burn scars were imaged with the QuantifiCare LifeViz Micro 3D camera. Three experienced wound care therapists first estimated, then measured using 3D Track software, the imaged scars’ height and volume. Two-factor analysis without replication was performed to calculate intraclass correlation coefficients (ICCs) between assessors’ estimated scar height and volume, and measured height and volume. Two-sided Wilcoxon tests were performed comparing the mean estimated height and volume with the estimated and measured outputs. The estimated scar height’s ICC was 0.595, and for volume, it was 0.531. The measured scar height’s ICC was 0.933 and for volume, it was 0.890. The estimated and measured volume were significantly different (z = −2.87, *p* = 0.041), while the estimated and measured height were not (z = −1.39, *p* = 0.161). Stereophotogrammic measurement of scar height and volume is more reliable than clinical photograph assessment. Stereophotogrammetry should be utilized when assessing burn scar height and volume, rather than subjective estimates from clinical scar tools.

## 1. Introduction

Hypertrophic scarring is indicative of the post-burn burden of disease, with the majority of burn patients developing this dermatoproliferative disorder [1]. Presenting as raised, painful, red, pruritic, and contractile, hypertrophic scars are the result of an exuberant and abnormal wound healing process [2]. The clinical phenotypes of hypertrophic scar are reflective of deviant cellular and molecular skin composition resulting from this abnormal healing. For example, increased water retention due to glycosaminoglycan content accounts for the positive volume of hypertrophic scars, and increased vascularization of the tissue is responsible for scar erythema [3,4]. Spontaneous attenuation of scar features occurs as the scar ages and is thought to cease when the scar is completely ‘mature’; at the point of maturation, the scar will no longer naturally regress, nor will therapeutic manipulation yield as substantial improvements as seen prior to maturation. Despite a reduction in the severity of burn scar features over time, the scar is often noticeably different from uninjured skin. The functional and cosmetic inadequacies of the post-burn scar have ramifications for patient recovery after their injury—worse scarring is associated with poor patient quality of life and depression [5,6]. As such, an antecedent to improved patient quality of life related to scar quality is enhanced burn scar assessment, such that the state and progression of wound healing can be elucidated, informing clinical initiative and treatment evaluation.

Numerous scar scales have been applied in clinical and research practice, namely the Vancouver Scar Scale (VSS) and the Patient and Observer Scar Assessment Scale (POSAS) [7,8]. While the VSS considers only clinician assessment of scar variables including pigmentation, vascularity, height, and pliability, the POSAS takes into consideration patient and clinician opinion of the scar, enabling evaluation of patient-reported pruritus and pain in addition to pliability, relief, thickness, vascularity, and pigmentation. In both research and clinical settings, standardization of burn scar assessment and consistency between users is integral for accurate clinical assessment and use of scar characteristics as clinical trial measures. Presently used scar scales do attempt to standardize scar assessment, but these subjective tools’ poor reliability limits their efficacy [9,10,11]. Discrepancies between POSAS patient and observer scores have also been described [12]. Apart from variability between users, scar scales are seldom validated for sensitivity to change over time, and such validations demonstrate weak correlations between subjective and objective determination of scar quality longitudinally [13]. While modifications to existing scar scales have attempted to mitigate these scales’ shortcomings, objective, quantitative measures of scars have the potential benefit of more accurately and reliably evaluating burn scar features, and therefore, scar evolution, treatment impact, and applicability for use in clinical trials [9,10,11,14].

Objective devices for measuring scar color, thickness, elasticity, and volume have been described [15,16,17,18]. While characteristics of color, thickness, and elasticity have been integrated into subjective scar evaluation, positive scar volume has been largely ignored in scar assessment. Although scar volume may spontaneously regress during maturation, the persistence of raised scars exceeding 30 years post-burn suggests that this aspect of the burn scar is clinically relevant when monitoring wound healing and scar quality during patient recovery [19]. Indeed, scar therapy studies view leveling of the scar as an indicator of treatment effectiveness, aiming to approximate the planar appearance of normal skin [20]. Instrumented efforts to measure scar tissue thickness and volume have implemented high-frequency ultrasound techniques [9,10,11,17,18]. However, poor correlation between the gold standard (i.e., biopsy and histological quantification) and ultrasonic determination of skin thickness has been reported, in addition to difficulty in achieving the necessary depth of tissue penetration with ultrasound technology [17]. As well, ultrasound equipment is comparably large, labor-intensive, and requires direct contact with the patient or research participant, necessitating concern for adequate decontamination between uses. The required user training for ultrasound lends itself to potential variability in measurement collection. A scar assessment modality that shows improvement from the constraints of current clinical tools for scar evaluation should account for volumetric measurements in addition to those traditionally captured during clinical interactions (such as color), as well as demonstrate reliability and accuracy through easy-to-use objective methodology. A description of surface ‘roughness’ is also clinically relevant, but as of yet there is no acceptable measure for this scar characteristic.

**Figure 1:**
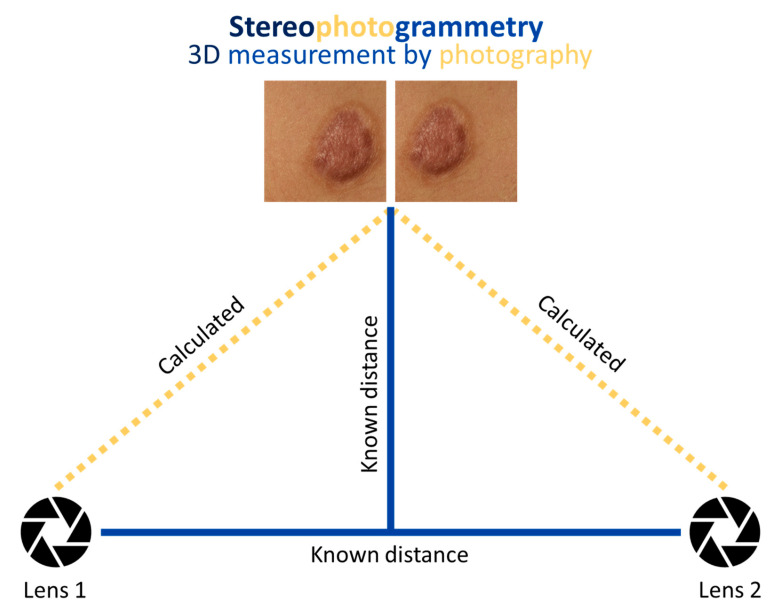
A stereophotogrammic approach involves taking two photographs of an object (i.e., burn scar) from differing angles, such that the two photographs overlap partially. In modern stereophotogrammetry, a double lens is appended to a digital camera for simultaneous image capture. Known distances in the object–camera system permit calculation of the object’s 3D coordinates by triangulation. The rendered 3D image can be measured using software.

Stereophotogrammetry, a non-invasive three-dimensional (3D) imaging and measurement technique, may be an appropriate tool for the quantification of burn scar characteristics. Stereophotogrammic technology has evolved from cumbersome imaging set ups to handheld, portable digital cameras, making it an attractive candidate for inclusion in clinical and research-based assessment of burn scars [21,22,23,24]. A stereophotogrammic approach involves taking two photographs of an object from differing angles wherein the images partially overlap (Figure 1) [25]. Corresponding coordinates between the two images and the known distance between the object camera system permit calculation of the image’s 3D coordinates and subsequent 3D image rendering [25]. From the generated 3D image, software analysis enables objective measurements. Stereophotogrammetry has been incorporated into the assessment of scars and wounds [21,22,23,24,26,27,28]. Where previous work has simply validated stereophotogrammetry as an accurate, reliable non-contact method to determine burn scar volume using patient and simulated scars, the applications of stereophotogrammetry in wound progression and non-burn scars have been more extensively explored [21,22,24,26,27,28]. Xu et al. utilized longitudinal measurements of wound parameters to develop individualized, predictive visualizations of healing in chronic wounds, suggesting the clinical importance of longitudinal monitoring using stereophotogrammetry [27]. Stereophotogrammic volume measurements have been further employed to address keloid response to intralesional steroid treatment successfully [28]. As such, the high clinimetric validity of stereophotogrammetry, coupled with its broad scope of application, implicates stereophotogrammetry as a valuable scar assessment modality with the potential to enhance scar assessment and management.

We have previously described preclinical assessment of stereophotogrammetry as a valid measure of burn-related scars [22]. The primary purpose of this study was to build upon the established validity of stereophotogrammetry by comparing the estimated versus instrumented measurement of maximum height and total positive volume of hypertrophic burn scars in a tertiary care adult outpatient burn clinic. The secondary purpose of this study was to evaluate the results and methods to prepare for a follow-up longitudinal multimodal, instrumented cohort research study to describe the evolution of post-burn hypertrophic scars.

## 2. Materials and Methods

This study was approved by our university’s Conjoint Health Research Ethics Board at the University of Calgary (REB21-0451); all participants provided written informed consent and stored in compliance with our institutional requirements. Enrollment in this study required that patients were 18 years of age and above and present at an outpatient burn clinic with closed burn scars that could be captured in a single image. For patients with numerous scars, each scar was photographed individually. Patients with scars from non-burn injuries or who were unable to provide consent were excluded. Information pertaining to patient characteristics, including the date of injury, date of image capture, age at consent, anatomic region of injury, and acute treatment of the wound, were collected from patient injury records. A sample size of 26 burn scars was obtained to replicate the sample size of our previous study evaluating stereophotogrammic assessment of scars [22]. No research participants withdrew from the study, and no adverse events occurred during the project.

The LifeViz Micro (QuantifiCare, Biot, France) is a portable, commercially available stereo camera that was used to photograph patients’ burn scars. Prior to use, the 3D camera was calibrated by photographing a standardized image provided by the manufacturer; the manufacturer then confirmed the system’s settings. The camera has a field size of approximately 7 × 9 cm, enabling image capture of scars within this area. To ensure reproducibility and the correct distance between the camera and photographed surface for 3D rendering, the LifeViz Micro projects two beams of light onto the surface to be photographed. When the user is at an appropriate distance perpendicular to the surface, the light beams will converge into a single beam, and image capture can commence.

Patients were photographed in the burn clinic, with consistent lighting and temperature. Flash photography was also used when imaging all scars to standardize light exposure conditions and allow surface detail to be captured. Patients were asked to remove pressure garments for a non-specified period of time to enable visualization of their scar(s). As this project a single image capture event, patients with previous or planned scar interventions were not excluded. Patients were positioned at their comfort, with a standard green surgical drop cloth placed behind the anatomy to be imaged. Images were obtained by one clinician during patients’ routine follow-up visits to the outpatient burn clinic.

**Figure 2:**
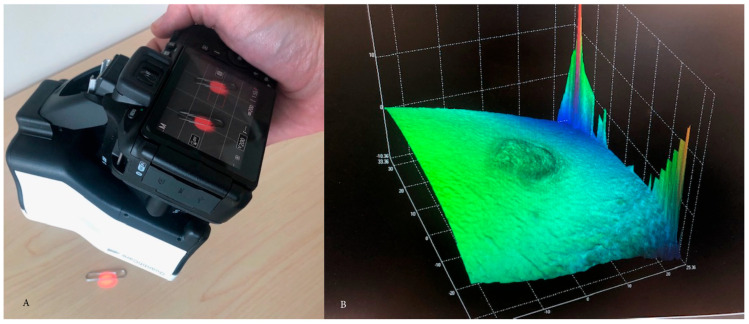
(**A**) Image capture, (**B**) 3D rendering of scar.

After image capture, photographs of scars were uploaded from the LifeViz Micro to a clinic desktop computer updated with additional processing power available through the hospital system to accommodate the stereophotogrammic software: 3D Track v6.18 (QuantifiCare, Biot, France). Images imported to the 3D Track interface immediately undergo 3D reconstruction, and the produced 3D image is then available for assessment within the software. No Wi-fi nor cellular connectivity was incorporated into the information transfer between devices. Standard 2D planar clinical photographs were taken and incorporated as usual clinical practice. To facilitate measurements of an image, the scar’s boundaries must be manually demarcated. Application of a closing surface to the image assists demarcation and provides a reference surface from which measurements based on the image’s calculated 3D coordinates can be made. Following the definition of the scar borders, the software provides measurement outputs relative to the contoured area. Measurements can be exported from 3D Track to an Excel file. Three experienced wound care therapists estimated the maximum height and total positive volume of the collected images. First, the assessors subjectively estimated maximum height and positive volume of each imaged scar. Following subjective estimates, assessors measured each photographed scar’s maximum height and positive volume in 3D Track. Figure 2A shows image capture and Figure 2B creation of digital mesh for measurement.

Results from image assessment and patient characteristics were exported to Excel v16 for further analysis. Two-factor analysis without replication was performed to calculate intraclass correlation coefficients (ICCs) between the assessors’ estimated scar height and volume and the measured height and volume. Two-sided Wilcoxin tests were performed comparing mean estimated height and volume between estimated and measured output.

## 3. Results

Twenty-six hypertrophic scar images were collected from fifteen patients enrolled in this study. Patient age ranged from 20 to 68 years (mean = 42.6, SD = 15.8). Patients’ acute burn wounds were most often managed by nonoperative treatment (*n* = 20); excision and grafting (*n* = 5) were less frequent (Table 1). A single scar image was taken from a skin graft donor site. Scars located on the trunk and extremities, but none on the head nor neck, were photographed.

Table 2, Table 3 and Table 4 showcase estimated and measured height and volume across assessors. Table 5 indicates the results of this study. Measured scar height showed greater interrater reliability (ICC = 0.933) than measured volume (ICC = 0.890), although both measured ICCs were well above those of estimated scar height (ICC = 0.595) and volume (ICC = 0.531). Comparison of assessors’ estimated and measured height and volume was achieved by Wilcoxon tests. Where estimated and measured volume were significantly different (z = −2.87, *p* = 0.041), estimated and measured height were not (z = −1.39, *p =* 0.161). 

## 4. Discussion

Stereophotogrammetry offers accuracy and sensitivity, as well as reliability to measurements while being an intuitive camera–software system setup [21,22,23,24]. Here, we demonstrated the benefit of stereophotogrammetry over the standard scar assessment practice, clinician estimate, in determining burn scar maximum height and total positive volume. The interrater reliability of stereophotogrammetry for volumetric measurements in burn scars has been previously documented, and our work adds to this with a comparison of scar volume and height obtained by clinician estimate and stereophotogrammic measurement [21,22]. Our findings suggest stereophotogrammic measurement of scar height and volume to be more reliable than clinical estimation from the same images. Estimation of scar volume from photographs is significantly less than instrumented measurement, although the maximum estimated vs. measured scar height was not significant in this study. Given the heterogeneity both within and between scars, the high interrater reliability of measured height and volume is appreciable and highlights the promise of stereophotogrammetry as an ideal clinical tool. We recommend stereophotogrammetry for integration into clinical and research environments assessing burn scar features for clinical follow-up and the effectiveness of burn scar treatment for height and volume. However, this technology should complement, not replace, subjective descriptions of scars to capture important scar variables of itch and pain that are not yet measurable by objective tools.

The inclusion of two-dimensional photography in clinical interactions is standard practice in burn care. So, incorporating instrumented scar assessment based on 3D photography into burn clinics and the eventual replacement of 2D clinical photography should be feasible. Modern stereophotogrammetry is rapid (with additional software analysis being more time-consuming than image capture), objective, intuitive, and non-invasive [21,22]. The compactness and portability of commercially available stereo cameras like the LifeViz Micro lend themselves well to clinical and research settings, though stereophotogrammetry is not without challenges. Scars of restricted dimensions can be captured by stereophotogrammetry in a single image—the field size of the LifeViz Micro prevents imaging of scars greater than 7 × 9 cm. Additionally, it may be difficult to adequately photograph burn scars on contoured surfaces of the body since curvature may hide regions of the scar [23]. Considering the advantages of stereophotogrammetry, these drawbacks do not inhibit the technology’s utility, and they are solvable. While not ideal, limitations of stereophotogrammetry can be overcome. As taking multiple single-frame images may not sufficiently address the issue of capturing body curvature, surface scanning with a multi-frame device may be better for larger and/or contoured scars, though at increased cost and image capture time.

The reliable and accurate assessment of burn scar volume by stereophotogrammetry is especially significant as current clinical descriptions of burn scars do not include volume. Various non-burn-related wound and scar studies have showcased the value of volumetric measurement by stereophotogrammetry, utilizing this measure and other scar parameters to indicate wound progression and scar therapy impact. In keloids, Ardehali et al. used stereophotogrammetry to quantify scar volume before and following regular intralesional steroid injections [28]. Monitoring the effectiveness of scar treatment via stereophotogrammetry may help improve outcomes by distinguishing unresponsive from responsive patients early on, which may, in turn, reduce patient burden of continuing with ineffective or multiple therapies and enable optimization of treatment strategies [28]. Further, stereophotogrammetry was able to differentiate changes in wound variables (including volume) over time [26]. Recognizing the technology’s ability to distinguish healing and non-healing wounds through the quantification of wound variables, Xu et al. developed a predictive model using stereophotogrammic measurement that generated individual healing curves for patients with pressure ulcers [27]. While the ability to identify wound healing in real-time is, in itself, valuable, these healing curves give clinicians the ability to predict healing. Images generated by stereophotogrammetry may also provide an avenue for patient–provider interactions, whereby patients can visualize changes in their scar’s 3D properties over time, facilitating enhanced patient understanding of their scar’s progression or response to treatment.

Analogous studies in burn scars that objectively document the evolution of scar geometry do not exist. Our follow-up study to this work is a long-term cohort study that will lead to the development of a novel, long-term human scar database. Single-frame stereophotogrammetry will be one technique used to collect parameters of burn scars over time, which will be instructive in creating predictive models for scar development. To facilitate scar relocation, planar imaging and transparent film marked over the measurement sites will be utilized along with image superimposition to validate positioning. For this ongoing work, stereophotogrammetry, as described in this project, offers accessible instrumented measurement of some physical characteristics of hypertrophic scars with the limitations of field size and normally occurring curvature. To address these issues for larger scars, we are incorporating structured light 3D scanning to capture scars that extend beyond the camera used in this project. Structured light 3D scanning cameras are currently significantly more expensive and require additional computer processing capability than the stereophotogrammetry camera. However, additional image characteristics such as texture or color may allow for a detailed description of scar appearance, and greater resolution will create a discrete digital image of the scar surface. Furthermore, the scar surface roughness is not described in this stereophotogrammetry project. However, we aim to devise means to create an assessment tool from structured 3D scanning images, since roughness in scars or that seen in meshed skin grafting presents a challenge to our current work, along with standardizing a method to establish a bottom reference stage to calculate volume from scans taken of complex anatomic regions. With these imaging techniques combined with complementary mechanical testing, we hope to create a longitudinal database for scar researchers.

This chronology of scar evolution is an important consideration when evaluating the outcomes of clinical research. For example, if it is accepted that there is a volume peak and regression over time of hypertrophic scars, interventions initiated and outcomes measured on the upward slope of volume may falsely report no efficacy when, in truth, the peak of scar volume may have been suppressed. Conversely, therapies applied on the downward slope of volume may demonstrate benefits when no difference from natural evolution was demonstrated. Investigational therapies would best address these issues if scar studies could be performed using within-scar controls; however, this may not be feasible due to the unknown effect spread within scar tissue of an applied therapeutic, impracticality of application such as for external pressure devices, or an unwillingness of researchers or participants to enroll in such clinical trials.

## 5. Conclusions

The disadvantages of clinician estimation of burn scar characteristics by subjective scales may be overcome with instrumented assessment of scars. Stereophotogrammetry shows increased reliability in the evaluation of burn scar maximum height and total positive volume. Stereophotogrammetry may be suitably employed in clinical and research contexts, considering the already standard practice of two-dimensional wound photography and its ease of use, to describe scar evolution and potential responses to interventions.

## Figures and Tables

**Table 1:** Patient (*n* = 15) and hypertrophic scar wound site (*n* = 26) characteristics. Scars were photographed on the trunk and extremities, but not on the head nor neck.

Characteristic	Patient Summary
Patient age, *n* = 15 (years)	20–68 (mean = 42.6, SD = 15.8)
Acute burn wound management, *n* = 26 (count)	
Non-operative treatment	20
Excision and grafting	5
Skin graft donor site	1

**Table 2:** Assessor 1 raw data from estimated and measured image assessment.

Image Number	Estimated Height (mm)	Estimated Volume (cm^3^)	Measured Height (cm)	Measured Volume (cm^3^)
1	2	0.02	0.06	0.25
2	2	0.32	0.03	0.14
3	3	0.036	0.5	0.19
4	3	0.6	0.07	0.46
5	1	2	0.02	0.19
6	1	0.18	0.01	0.03
7	3	0.18	0.07	1.18
8	3	42	0.09	2.39
9	0	0	0.05	0.63
10	2	0.26	0.02	0.2
11	2	3.6	0.03	0.22
12	0.9	1.08	0.07	1.01
13	0.05	0.4	0.11	1.02
14	1	4.5	0.06	0.22
15	1	2.5	0.06	0.38
16	1	2.45	0.05	0.61
17	3	4.5	0.06	0.4
18	3	0.675	0.09	0.53
19	1	3.15	0.11	0.89
20	0.2	2	0.11	1.49
21	0.1	1.5	0.15	2.42
22	0.5	2.1	0.11	0.5
23	4	12.6	0.27	3.65
24	3	7.5	0.1	1.94
25	1	2.1	0.7	0.23
26	3	9.45	0.14	4.19

**Table 3:** Assessor 2 raw data from estimated and measured image assessment.

Image Number	Estimated Height (mm)	Estimated Volume (cm^3^)	Measured Height (cm)	Measured Volume (cm^3^)
1	3	0.234	0.06	0.14
2	2	0.4	0.03	0.05
3	2	0.48	0.05	0.18
4	3	0.504	0.06	0.13
5	2	0.4	0.03	0.14
6	2	0.09	0.02	0.01
7	2	2.1	0.07	0.88
8	3	2.02	0.1	2.08
9	0.5	4.625	0.05	0.86
10	0.2	0.48	0.03	0.52
11	2	13.12	0.04	0.83
12	0.5	1.8	0.11	1.02
13	2	2.1	0.11	1.01
14	2	0.304	0.06	0.18
15	1	3.08	0.06	0.4
16	3	0.576	0.02	0.13
17	3	0.72	0.07	0.5
18	2	0.14	0.05	0.12
19	3	0.6	0.13	1.02
20	0.5	4.75	0.12	0.02
21	1	2.5	0.13	1.78
22	3	0.66	0.08	0.19
23	4	7.56	0.23	2
24	3	5.25	0.09	1.57
25	2	0.084	0.02	0.08
26	2	1.79	0.1	2.69

**Table 4:** Assessor 3 raw data from estimated and measured image assessment.

Image Number	Estimated Height (mm)	Estimated Volume (cm^3^)	Measured Height (cm)	Measured Volume (cm^3^)
1	2.5	0.75	0.06	0.15
2	2	0.36	0.03	0.14
3	4	1.44	0.05	0.17
4	1.5	3.75	0.07	0.17
5	1	0.5	0.03	0.18
6	1	0.04	0.01	0.02
7	1	1.2	0.07	0.98
8	2	3.6	0.08	1.83
9	1	0.9	0.06	0.82
10	0.5	3	0.02	0.18
11	2	2.5	0.03	0.24
12	0.2	0.16	0.05	0.62
13	2	1.5	0.11	1.02
14	1.5	0.225	0.06	0.17
15	1	1.2	0.06	0.31
16	1	0.525	0.05	0.51
17	3	0.078	0.07	0.36
18	3	0.225	0.04	0.17
19	1	0.525	0.11	0.91
20	0.5	7.5	0.1	1.08
21	0.5	7.5	0.14	2.31
22	0.75	2.34	0.1	0.38
23	5	16.25	0.25	2.86
24	3	6.75	0.09	2.04
25	0.5	0.937	0.02	0.15
26	1.5	3.937	0.13	3.89

**Table 5:** Interrater reliability and comparison of estimated vs. measured height and volume. ICC, intraclass coefficient.

Burn Scar Characteristic	ICC	Z-Score (*p*-Value)
Maximum height		−1.39 (0.161)
Estimated	0.595	
Measured	0.933	
Positive volume		−2.87 (0.041)
Estimated	0.531	
Measured	0.890	

## Data Availability

Original data is available by request from the corresponding author.
